# A Role for RAGE in DNA Double Strand Breaks (DSBs) Detected in Pathological Placentas and Trophoblast Cells

**DOI:** 10.3390/cells10040857

**Published:** 2021-04-09

**Authors:** Kary Y.F. Tsai, Benton Tullis, Katrina L. Breithaupt, Rylan Fowers, Nelson Jones, Samuel Grajeda, Paul R. Reynolds, Juan A. Arroyo

**Affiliations:** Lung and Placenta Research Laboratory, Department of Physiology and Developmental Biology, Brigham Young University, Provo, UT 84602, USA; karyyftsai@gmail.com (K.Y.F.T.); bentontullis@yahoo.com (B.T.); klbreit7@gmail.com (K.L.B.); fowersrylan@gmail.com (R.F.); Nelsonmrjones@gmail.com (N.J.); grajeda.samuel@gmail.com (S.G.); paul_reynolds@byu.edu (P.R.R.)

**Keywords:** DSB, RAGE, placenta, γ-H2AX, pATM, MRE11

## Abstract

Impaired DNA damage responses are associated with several diseases, including pregnancy complications. Recent research identified an ATM-kinase dependent function for the nuclear isoform of the receptor for advanced glycation end-products (RAGE) during double strand break (DSB)-repair. RAGE contributes to end-resectioning of broken DNA sites by binding with the MRE11-Rad50-Nbs1 (MRN) complex. Placental research is limited regarding the impact of genomic instability and the mechanism for potential repair. We tested the hypothesis regarding the involvement of RAGE during the repair of placental DNA-DSBs. We first identified that the pregnancy complications of PE and preterm labor (PTL) experience loss of genomic integrity and an in vitro trophoblast cell model was used to characterize trophoblast DSBs. Colocalized immunofluorescence of γ-H2AX and RAGE support the potential involvement of RAGE in cellular responses to DNA-DSBs. Immunoblotting for both molecules in PE and PTL placenta samples and in trophoblast cells validated a connection. Co-immunoprecipitation studies revealed interactions between RAGE and pATM and MRE11 during DNA-DSBs. Reduced cellular invasion confirmed the role of genomic instability in trophoblastic function. Collectively, these experiments identified genomic instability in pregnancy complications, the impact of defective DNA on trophoblast function, and a possible RAGE-mediated mechanism during DNA-DSB repair.

## 1. Introduction

The placenta is a critical organ during pregnancy, serving as the maternal-fetal interface. Throughout pregnancy progression, fetus-derived trophoblasts play key roles in establishing and maintaining placental function and growth [1]. Early in pregnancy, trophoblasts invade the uterine endometrium and myometrium, and convert resident spiral arteries from high to low resistance vessels, in order to increase blood capacity [1]. Besides the importance of invasion, trophoblast cells are also specialized epithelial cells that are responsible for facilitating appropriate exchange of nutrients, and wastes between maternal and fetal compartments [2]. These unique functions allow embryonic settlement and meets the requirement of enhanced oxygen and nutrient exchange for proper fetal development [3]. Abnormal placentation is a feature of diverse pregnancy complications including pre-term labor (PTL), intrauterine growth restriction (IUGR) and preeclampsia (PE) [4,5,6]. Failures in placental formation result from inappropriate adaptation, shallow trophoblastic invasion, increased trophoblast apoptosis, and insufficient spiral artery modification compromise placental and embryonic growth, and development [7,8]. Therefore, trophoblast survival and invasion are essential for successful pregnancies, whereas trophoblast apoptosis and dysfunction correlate with pregnancy complications, and such outcomes may be underpinned with placental genomic instability [9].

DNA damage can be a part of the natural progression of the cell cycle and a means of disposing aberrant cells [10,11]. However, exposure to cytotoxic agents also results in defective DNA accumulation and negatively affects physiological function. The most cytotoxic forms of DNA damage are DNA double-strand breaks (DNA-DSBs) where both strands of the helix are ruptured. If left unrepaired, these lesions lead to disorders such as cancer, fibrosis, and neurodegeneration [12,13]. A functional repair system is fundamental in maintaining human health when the harmful effects of DNA-DSBs are manifest. In response to DNA-DSBs, sequential events involved in the DNA damage response (DDR) will detect, recruit various implicated molecules, and repair DNA-DSBs via either the error-prone non-homologous end joining (NHEJ) pathway or the error-free homologous recombination repair (HRR) [11,12,13]. Specifically, during HRR, DSBs activate ataxia-telangiectasia-mutated (ATM), which functions as the central controller of cellular responses to DNA damage [14]. ATM subsequently phosphorylates several downstream effectors, including the activated histone variant γ-H2AX, which marks the locations of DSBs, and the DNA damage sensor MRE11-Rad50-Nbs1 (MRN) complex, which is essential at damaged DNA sites for end processing [15].

The receptor of advanced glycation end products (RAGE) is a multi-ligand receptor primarily expressed on cell membranes [16]. Common ligands for RAGE include advanced glycation end-products (AGEs) and high mobility group box 1 (HMGB1). The RAGE-ligand interaction is well-recognized for its modulation of chronic inflammatory diseases, such as type 2 diabetes, varieties of neurodegeneration, and chronic obstructive pulmonary disease [17,18]. More recently, our laboratory showed that increased placental expression of membrane bound RAGE contributes to low trophoblastic survival and insufficient invasion in a model of IUGR, suggesting a role of RAGE during placental disease [19]. Although membrane bound RAGE is predominately known for its function in inflammatory signaling, the nuclear isoform of RAGE (~64 kDa) was recently identified and demonstrated to be a positive regulator during DNA-DSB repair in the lungs via HRR. During HRR events, RAGE is activated by DSB-induced ATM kinase and function with MRN complexes in facilitating end-resectioning at the broken sites of DNA [18].

Although the accumulation of DNA damage has been observed in pathological placentas, knowledge regarding the impact of genomic instability on pregnancy complications is still limited [20]. The current research sought to, (1) identify pregnancy complications that are associated with genomic instability, (2) understand the effects of DNA damage and its impact on trophoblast invasion, and (3) investigate the functional requirements of RAGE during placental DNA-DSB sensing. Collectively, our study provides novel insights into DNA damage-associated pregnancy complications and identifies the physiological relevance of molecules in the repair process that may foreshadow the development of pregnancy complication treatments.

## 2. Materials and methods

### 2.1. Human Placental Tissues

All placental biopsies and slides from paraffin embedded placental tissues (gestational diabetes (GDM), preterm labor (PTL), preeclampsia (PE) and term control (Cntl) were purchased from the Research Center for Women’s and Infant’s Health BioBank, Ontario, Canada. In total, there were 6 samples analyzed for each control and disease group. Samples were collected from placentas, delivered in conjunction with delivery of the fetus, either vaginally or by C-section. Sample demographics are shown in Table 1.

### 2.2. Cell Culture and Treatments

The first trimester trophoblast cell line, Sw.71, and the choriocarcinoma cell line, Bewo, were used for these studies (*n* = 10; *n* = number of experiments performed in triplicate). Both lines were cultured in appropriate cell culture medium (Sw.71, RPMI; Bewo DMEM/F-12) supplemented with 10% fetal bovine serum (FBS)and 1% penicillin and streptomycin. DNA-DSBs were induced by treating cells with 1% cigarette smoke extract (CSE) for 24 h., or 30 μg/mL of Bleomycin (BLM; a commonly used chemotherapy drug for cancer treatment known to induce DSBs.) for 1 h [21,22]. A neutralizing RAGE antibody (nAb; 2.4 μ/mL), was used in RAGE targeting studies, which blocks functional RAGE via recognition of a 300-residue sequence of the receptor’s extracellular domain.

### 2.3. Cigarette Smoke Extract (CSE)

Nicotine is a key component in cigarette smoke that is associated with the development of DBS [23]. Preliminary studies in our laboratory (data not shown) showed nicotine to be the main component of CSE. CSE was generated, as previously described by Lewis et al. (2017) [19]. Briefly, one 2RF4 research cigarette (University of Kentucky, Lexington, KY, USA) was continuously smoked with a vacuum pump into 5 mL of RPMI or DMEM/F-12 medium (Mediatech, Manassas, VA, USA). The smoke-bubbled medium was filtered through a 0.22-μm filter to remove large particles. The resulting medium was defined as 100% CSE and dilutions were made using RPMI or DMEM/F12 medium to a starting stock concentration of 20% CSE. CSE was made fresh for every treatment.

### 2.4. Immunofluorescence (IF)

Table 2 lists the antibodies used in these experiments. IF was performed on paraffin embedded placental sections (*n* = 6; *n* = number of placental sections per condition) or trophoblast cells (*n* = 6; *n* = number of experiments conducted in triplicate) as previously performed in our laboratory [24]. Briefly, serial sections were incubated overnight with rabbit or mouse polyclonal antibodies against phospho-γ-H2AX (Cell Signaling, Danvers, MA, USA) or RAGE (R&D Technologies, Minneapolis, MN, USA). Anti-mouse fluorescein or Texas red-conjugated secondary antibodies were incubated for 1 h; 40,6-diamidino-2-phenylindole dihydrochloride (DAPI) was used for nuclear counterstaining. Slides were viewed on a BX61fluoresce microscope using the appropriate excitation and emission filter (fluorescein or rhodamine filters).

### 2.5. DNA Degradation

Genomic DNA samples were isolated using the genomic DNA mini kit (Thermo Scientific, Rockford, IL, USA, #K182002). Briefly, placental and cell samples (*n* = 6) were mixed with PBS, proteinase K, RNase A, and genomic lysis/binding buffer and incubated at 55 °C for 10 min for protein digestion. 96–100% ethanol was added to the mixture and the lysates were transferred to a spin column and spun down at 1000× *g* for 1 min. Spin columns were washed twice with washing buffer and the DNA were eluted by the elution buffer. Sample concentrations were tested using nanodrop and stored at −20 °C prior to applications. Following these steps, samples were electrophoresed on a 70% gel followed by a 1-h EtBr staining. The extent of DNA degradation was quantified using the Image Studio software (LI-COR Biosciences V5.2.5. Lincoln, NE, USA).

### 2.6. Cytoplasmic and Nuclear Extraction

Subcellular protein extractions (*n* = 10; *n* = number of extractions performed) were done by following the protocol included with the NE-PER Nuclear and Cytoplasmic Extraction Reagents kit (Thermo Scientific, Rockford, IL, USA #78835). Essentially, placental tissues or collected trophoblast cells were combined with CER I in microcentrifuge tubes and incubated on ice for 10 min. CER II was then added to the tubes and vortexed for 5 s, incubated on ice for 1 min, then vortexed again for 5 s. The tubes were then centrifuged at 16,000× *g* and at 4 °C for 5 min. Supernatants (cytoplasmic fraction) were collected into clean, pre-chilled, pre-labeled microcentrifuge tubes and stored in a −80 °C freezer before applications. The remaining pellets were re-suspended with ice-cold NER supplemented with 50 U/mL of Benzonase and sonicated for 5 times, 5 s each. Microcentrifuge tubes were again centrifuged at 16,000× *g* and at 4 °C for 10 min. Supernatants (nuclear fraction) were collected into clean, pre-chilled, pre-labeled microcentrifuge tubes and stored in a −80 °C freezer before applications.

### 2.7. Immunoprecipitation

CSE or BLM treated cells were lysed in RIPA buffer, supplemented with 50 U/mL Benzonase, and a cocktail of protease/phosphatase inhibitors. Lysates of 300 μg (*n* = 10; number of experiments performed) were pre-cleared with A/G- agarose beads and incubated with primary antibodies (phospho-(p)ATM-Cell Signaling Technology, Danvers, MA, USA, mouse#4526 or MRE11-Cell Signaling Technology, Danvers, MA, USA, rabbit#4895) overnight on a shaker at 4 °C. On the following day, 20 μg of A/G-agarose beads (Santa Cruz, CA, USA) were added to the mixture and incubated for 30 min at 4 °C on a shaker. The bead-protein complexes were collected by centrifugation and washed three times using RIPA buffer. 2× Laemmle buffer supplemented with DTT (total volume of 20 μg) were added to the collected pellets and incubated at 100 °C for 10 min to disassociate the beads and the attached proteins. The supernatant contents were then analyzed using western blotting.

### 2.8. Western Blotting

Placental tissues (*n* = 6; *n* = number of placental samples per condition) and cell lysates (*n* = 10; *n* = number of experiments performed; 10–20 μg) were separated on a 4–12% Bis-Tris gel and transferred to a nitrocellulose membrane. Membranes were incubated overnight with primary antibodies (phospho-γ-H2AX-Cell Signaling Technology, Danvers, MA, USA, rabbit #9718, RAGE-R&D, Minneapolis, MN, USA, goat #AF1145, p-ATM-Cell Signaling Technology, Danvers, MA, USA, mouse #4526, MRE11-Cell Signaling Technology, Danvers, MA, USA, rabbit #4895, β-Actin-Cell Signaling Technology, Danvers, MA, USA, rabbit #4967 or mouse #3700 and Lamin B1-Cell Signaling Technology, Danvers, MA, USA, rabbit#15068). The membranes were then incubated with fluorescent secondary antibodies for an hour and washed ×3 with TBST the next day prior to imaging.

Membranes were developed on a Li-COR Odyssey CLx. Fluorescence densities were determined, and comparisons were made between treated and control groups.

### 2.9. Real Time Cell Invasion

Real-time invasion of trophoblast cells (*n* = 10; *n* = number of experiments performed) was measured using xCELLigence RTCA DP (Real Time Cell Analysis Dual Plate) instrument from ACEA Biosciences Inc., San Diego, CA, USA on a 16 well CIM-Plate. These plates are composed of an upper and lower chamber, each containing 16 wells. The top wells were coated with Matrigel collagen and incubated for 4 h. Treated trophoblast cells (CSE or BLM) were plated in the top chamber at a concentration of 20,000 cells/well. The bottom chamber wells were filled with 10% FBS RPMI. The plates were then place in the RTCA DP instrument and invasion readings were taken every 15 min for 24 h.

### 2.10. Statistical Analysis

End-points obtained from DNA degradation studies and protein levels of (γ-H2AX, pATM, RAGE and MRN) were statistically compared. Differences in means ± SE were specifically assessed using the Mann-Whitney U-test. Significant differences between the groups were noted at *p* < 0.05.

## 3. Results

### 3.1. RAGE and Placental DNA-DSBs

**Figure 1:**
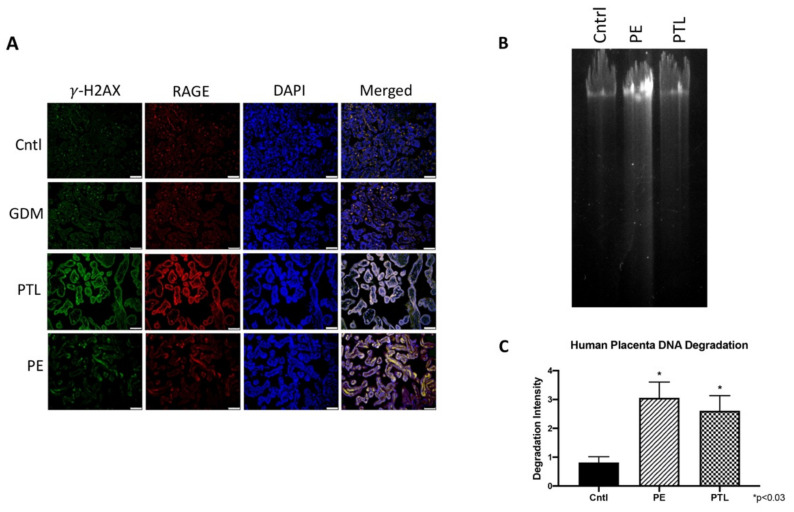
Genomic instability in pregnancy complications. (**A**) Increased staining and co-localization of DNA-DSBs (**Green**, γ-H2AX), and RAGE (**Red**) was observed during PE and PTL conditions. DAPI (**Blue**) was used for nuclear staining. (**B**) Gel electrophoresis tail length revealed the degree of DNA damage. From left to right: Cntl, PE, and PTL). (**C**) Quantifications of the fragmented DNA tail length from DNA electrophoresis (*n* = 6). Images (20× magnification) are representative of experiments involving at least 6 placental sections from each group.

When DNA-DSBs occur, such events are immediately followed by the phosphorylation of the histone octamer H2AX. This newly phosphorylated protein is the first step in the sequential process that leads to the identification of DSBs and activation of DDR, hence, γ-H2AX is a reliable marker for DNA-DSBs [25]. To determine the expression pattern of γ-H2AX and the functional relevance of RAGE, human placental samples were subjected to immuno-detection of γ-H2AX and RAGE. Qualitative immunofluorescence staining showed increased expression and colocalization of γ-H2AX and RAGE in the PTL and PE placentas when compared to Cntl (Figure 1A).

### 3.2. Genomic Instability in Pregnancy Complications

Timely repair of damaged DNA is essential in maintaining a healthy pregnancy, whereas defective DNA accumulation has been observed in pathological placentas [26]. To confirm the association of DNA damage in pregnancy complications, genomic DNA samples extracted from human placentas complicated by pre-term labor (PTL), preeclampsia (PE) and of normal gestation control (Cntl) were subjected to DNA degradation analysis via gel electrophoresis. Figure 1B depicts characteristic DNA degradation. Quantification of the resulting DNA degradation gel electrophoresis revealed elevated DNA fragmentation in the PE (3.8-fold; *p* < 0.01) and PTL (3.2-fold; *p* < 0.01) samples when compared to controls (Figure 1C). These data suggest the presence of DNA damage in pathological PE and PTL placentas.

### 3.3. RAGE and Placental γ-H2AX Nuclear Expression

**Figure 2:**
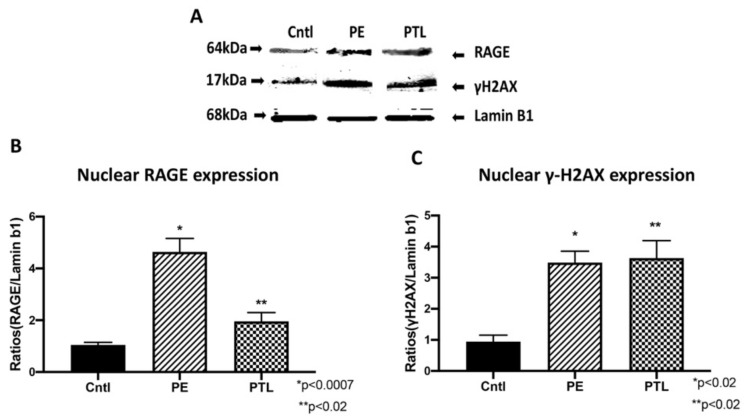
RAGE and placental γ-H2AX nuclear expression. (**A**) characteristic western blot for these experiments. (**B**,**C**) Elevated γ-H2AX and RAGE protein expression through western blot on PE and PTL samples when compared to controls.

After observing enhanced staining of RAGE and γ-H2AX in the PE and PTL placental tissues, we next investigated nuclear expression of these proteins in these diseased placentas and controls. A characteristic western blot for placental RAGE and γ-H2AX is shown in Figure 2. Immunoblotting of nuclear protein extractions from PE and PTL placental samples revealed increased nuclear RAGE (4.4-fold; *p* < 0.0007 and 1.9-fold; *p* < 0.02) and γ-H2AX (3.7-fold; *p* < 0.02 and 3.9-fold; *p* < 0.02) relative to normal gestation controls (Figure 2A,B).

### 3.4. RAGE Interacts with ATM and MRE11 during Placental DNA-DSBs

**Figure 3:**
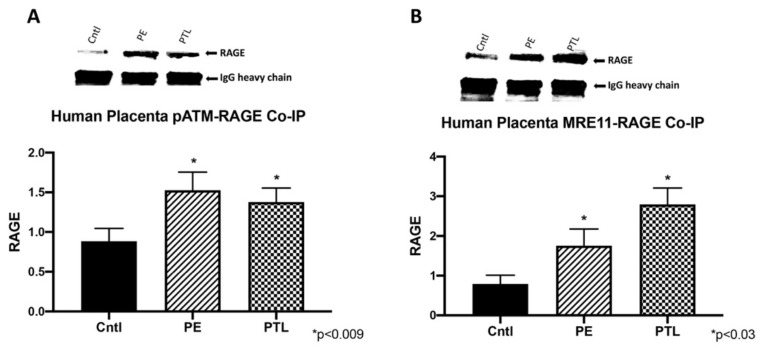
RAGE interacts with ATM and MRE11 during placental DNA-DSBs. (**A**) Increased pATM-RAGE complex in PE and PTL samples when compared to controls. (**B**) Increased MRE11-RAGE complex in PE and PTL samples when compared to controls.

Previous work showed the recruitment of activated RAGE by p-ATM to the site of DNA damage and RAGE modulation of MRE11 during DNA repair [18]. To implicate a similar role for RAGE during placental DNA-DSBs, ATM- or MRE11-RAGE interactions were verified by co-immunoprecipitation. In human placenta samples, immunoprecipitation of pATM showed expression of RAGE in the PE and PTL placentas (1.7-fold; *p* < 0.008 and 1.5-fold; *p* < 0.008; Figure 3A). Similarly, MRE11 complexed with RAGE was observed in PTL and PE (2.2-fold; *p* < 0.03 and 3.5-fold; *p* < 0.03; Figure 3B).

### 3.5. Role of RAGE in Trophoblast DNA Damage

**Figure 4:**
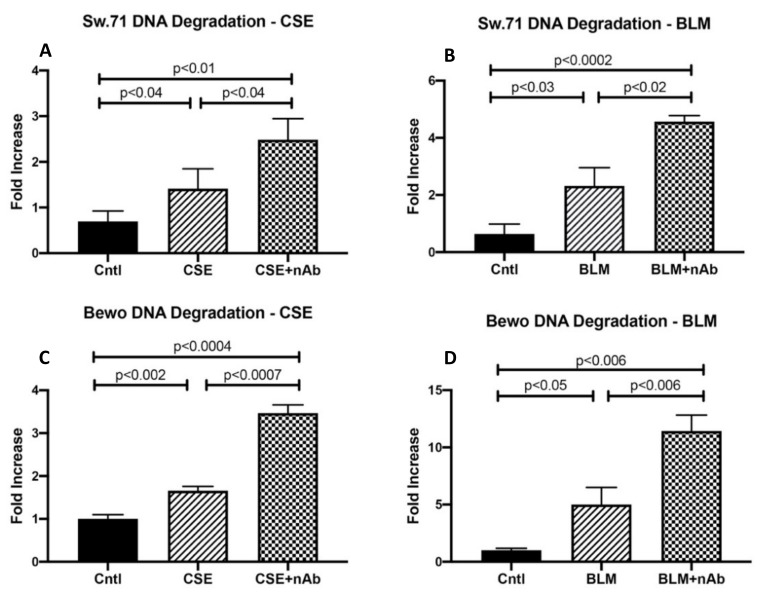
Role of RAGE in trophoblast DNA damage. (**A**) Increased DNA degradation in CSE treated Sw.71 cells. (**B**) Increased DNA degradation in BLM treated Sw.71 cells. (**C**) Increased DNA degradation in CSE treated Bewo cells. (**B**) Increased DNA degradation in BLM treated Bewo cells. (**A**–**D**), worsened DNA damage was present with the addition of neutralizing RAGE antibody in both cell types.

**Figure 5:**
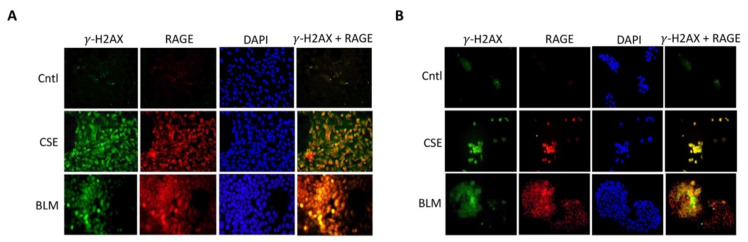
Trophoblastic DNA-DSBs and RAGE (IF). (**A**,**B**) Increased staining and co-localization of DNA-DSBs (**green**, γ-H2AX), and RAGE (**Red**) was observed from CSE or BLM treated Sw.71 (**A**) and Bewo (**B**). Images (40× magnification) are representative of experiments involving at least 6 different trophoblast experiments from each group.

To better understand RAGE function during DNA-DSBs, in vitro studies were performed with two placental trophoblast cell lines: Sw.71 (cytotrophoblast) and Bewo cells (syncytiotrophoblast). Exposure to tobacco smoke during gestation elicits numerous deleterious outcomes, and in particular, exposure has been linked to trophoblastic apoptosis, DNA damage and increased reactive oxygen species [27]. To understand the impact of cigarette smoke on DNA integrity of placental cell types, Sw.71 and Bewo were treated with cigarette smoke extract (CSE) or bleomycin (BLM; a known inducer of DSB) prior to assessing DNA damage. CSE and BLM treatment increased DNA degradation in both Sw.71 (2.0-fold; *p* < 0.05 and 3.6-fold; *p* < 0.05) and Bewo (1.6-fold; *p* < 0.003 and 5.0-fold; *p* < 0.05) cells when compared to the no treatment control cells (Figure 4A–D). To further elucidate the relevance of RAGE during DNA damage, a neutralizing RAGE antibody (nAb), which blocks functional RAGE, was used to treat cells in combination with CSE or BLM. When neutralizing RAGE was used in tandem with CSE or BLM, DNA degradation was further increased in Sw.71 (55%; *p* < 0.02 and 50%; *p* < 0.03) and Bewo (49%; *p* < 0.0004 and 44%; *p* < 0.006) cells as compared to cells treated with CSE or BLM alone (Figure 4A–D). In agreement with this DNA-DSB scenario, in vitro induction of DSBs using CSE or BLM in trophoblast cells led to increased co-expression of γ-H2AX and RAGE for both Sw.71 and Bewo cells as demonstrated by IF staining (Figure 5).

### 3.6. Trophoblast RAGE and γ-H2AX Nuclear Expression

**Figure 6:**
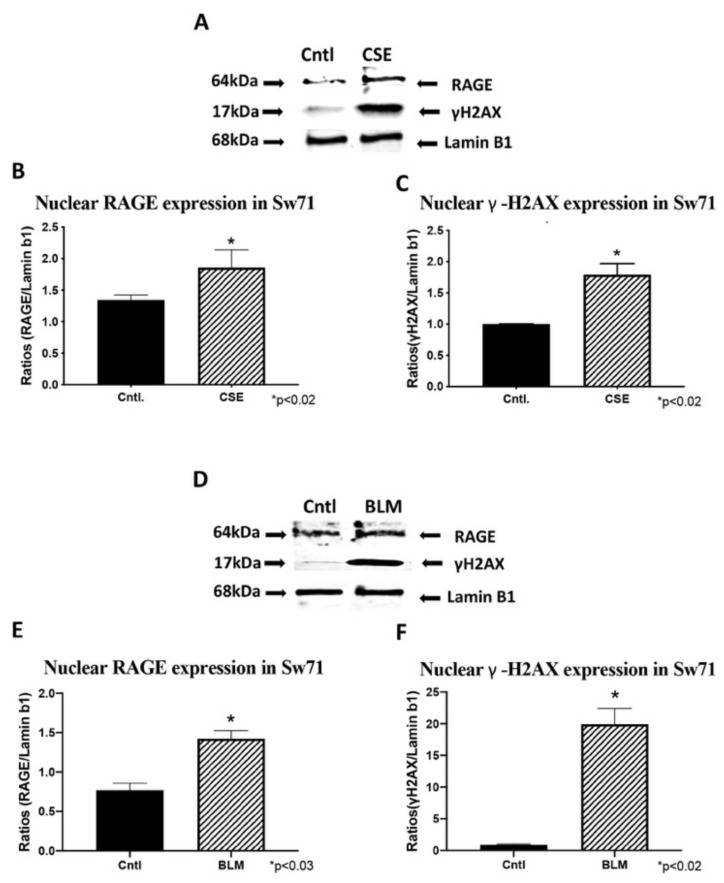
Trophoblast cell, Sw.71, DNA-DSBs and RAGE (WB). (**A**) Characteristic western blot for γ-H2AX and RAGE in CSE treated Sw.71 cells. (**B**,**C**) Elevated γ-H2AX and RAGE protein expression in CSE treated cell samples when compared to controls. (**D**) Characteristic western blot for γ-H2AX and RAGE proteins in BLM treated Sw.71 cells. (**E**,**F**), Elevated γ-H2AX and RAGE protein expression in BLM treated cell samples when compared to controls.

**Figure 7:**
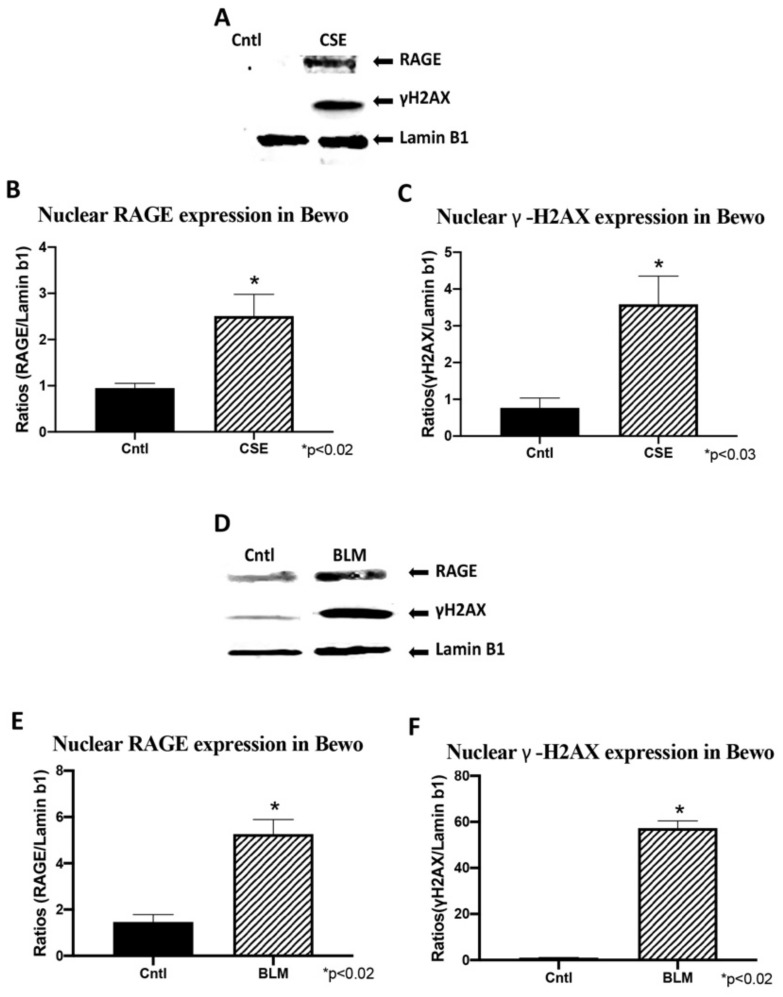
Trophoblast cell, Bewo, DNA-DSBs and RAGE (WB). (**A**) Characteristic western blot for γ-H2AX and RAGE in CSE treated Bewo cells. (**B**,**C**) Elevated γ-H2AX and RAGE protein expression in CSE treated cell samples when compared to controls. (**D**) Characteristic western blot for γ-H2AX and RAGE proteins in BLM treated Bewo cells. (**E**,**F**) Elevated γ-H2AX and RAGE protein expression in BLM treated cell samples when compared to controls.

To confirm RAGE and γ-H2AX nuclear expression, western blot was performed on nuclear fractions from trophoblast cells. CSE and BLM treatment of Sw.71 cells increased nuclear expression of RAGE (1.4-fold; *p* < 0.02 and 13.8-fold; *p* < 0.02; Figure 6A,C, respectively) and γ-H2AX (1.8-fold; *p* < 0.02 and 22.0-fold; *p* < 0.02; Figure 6B,D, respectively). Similarly, CSE and BLM treatment of Bewo cells increased both RAGE (2.6-fold; *p* < 0.02 and 3.6-fold; *p* < 0.02; Figure 7A,C, respectively) and γ-H2AX (4.7-fold; *p* < 0.03 and 54.8; *p* < 0.02; Figure 7B,D, respectively).

### 3.7. RAGE Interacts with ATM and MRE11 during CSE and BLM Induced DNA-DSBs

**Figure 8:**
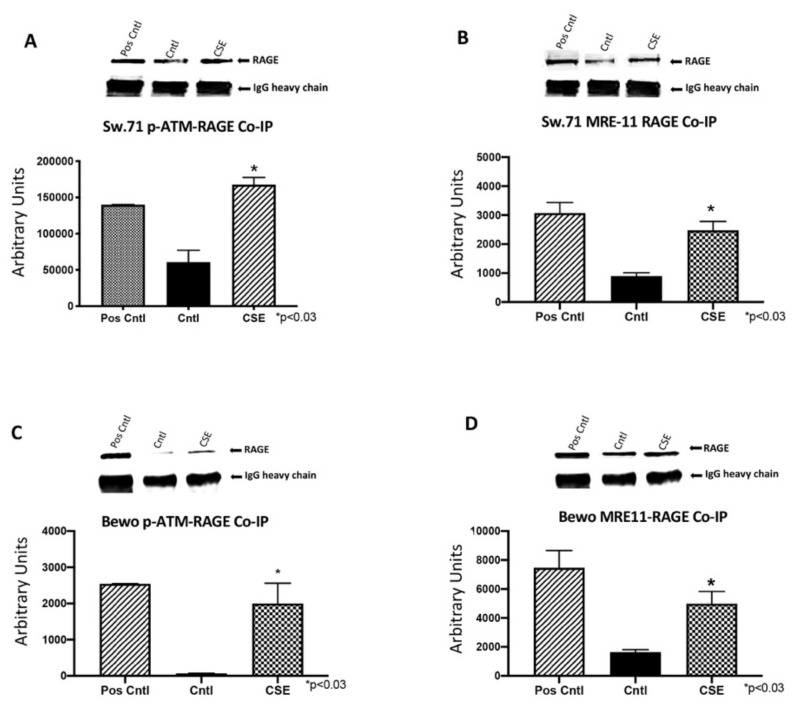
RAGE interacts with ATM and MRE11 during CSE and BLM induced DNA-DSBs. Increased pATM-RAGE (**A**) or MRE11-RAGE (**B**) complexes in CSE treated Sw.71 when compared to controls. Increased pATM-RAGE (**C**) or MRE11-RAGE (**D**) complexes in CSE treated Bewo cells when compared to controls.

DNA-DSBs induced in Sw.71 cells by CSE orchestrated the recruitment of RAGE by pATM (2.8-fold; *p* < 0.03; Figure 8A) and MRE11 (2.8-fold; *p* < 0.03; Figure 8B) to the site of DNA damage. CSE exposure also led to the recruitment of RAGE in Bewo cells by pATM (27.8-fold; *p* < 0.04; Figure 8C) and MRE11 (3.0-fold; *p* < 0.03; Figure 8D). These data identified RAGE as a probable substrate during the activation of placental DNA-DSBs repair programs.

### 3.8. Trophoblast Dysfunction as a Consequence of Genomic Instability

**Figure 9:**
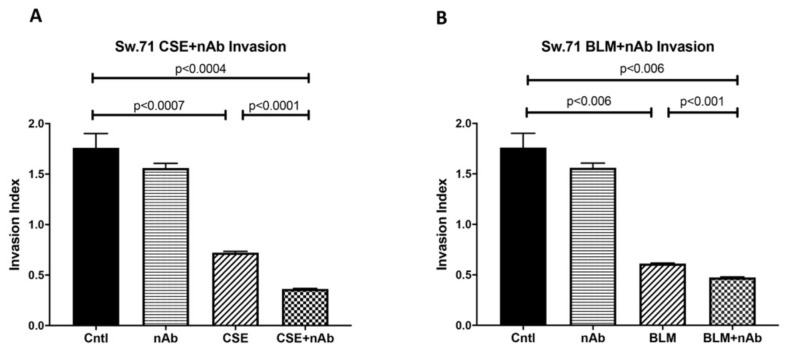
Trophoblastic dysfunction as a consequence of genomic instability. (**A**) Decreased invasion in CSE treated Sw.71 cells and further hindrance in invasion when nAb was co-treated with CSE. (**B**) Decreased invasion in BLM treated Sw.71 cells, the invasion further diminished when nAb was co-treated with BLM.

The crucial invading function of trophoblasts is essential for successful placental development. To determine the effects of DNA damage on trophoblast invasion, cell invasion assays were performed using the Sw.71 cell line treated with CSE or BLM and invasion was compared with the no treatment controls. Trophoblast invasiveness was decreased with CSE or BLM treatment (Figure 9A,B: 2.4-fold; *p* < 0.0007 and 2.9-fold; *p* < 0.006). Tellingly, RAGE nAbs administered with CSE or BLM further decreased invasiveness (Figure 9A,B: 4.9-fold; *p* < 0.0004 and 3.7-fold; *p* < 0.006). These results suggest that DNA instability compromises trophoblast invasion and contributes to anomalous pregnancy progression.

## 4. Discussion

DNA double strand-breaks (DNA-DSBs) are known as one of the most damaging forms of DNA complications as they can significantly compromise DNA integrity. Recently, a new role for RAGE was established during DNA-DSB recognition, as well as repair programs [18]. Previous reports showed increased DSBs in the PE placenta, but only scant reporting on such DNA defects exist for pregnancies complicated with PTL or GDM [24]. In the current research, we assessed the qualitative expression of γ-H2AX as a key marker of DNA-DSBs [15]. Detectable staining for γ-H2AX was observed in the PE and PTL placentas. This staining pattern correlated with RAGE localization in these pathological placentas as well. To our knowledge, this is the first report demonstrating DNA-DSBs in the PTL placenta, and the finding of a correlation of γ-H2AX and RAGE during PE and PTL obstetric complications. We further confirmed that both proteins were increased in the nucleus of trophoblast cells in both PE and PTL placentas. This is of interest as RAGE has classically been thought to be a membrane receptor and in our experiments, we observed potential tangential effects of RAGE in the nucleus and co-association with elevated nuclear expression of the DNA-DSB marker γ-H2AX [28,29,30]. To further confirm specific interactions of RAGE with DSB-associated complexes, immunoprecipitation was performed for pATM or MRE11, followed by RAGE protein detection. MRE11 and ATM are integral components of complexes that function in both the recognition of DNA-DSBs and repair programs, such as the preferred pathway involving homologous end joining [31,32,33]. MRE11 assembles into the MRN complex by binding with RAD50 and NBS1 proteins. MRN complexes rapidly recognize and localize to DNA-DSB foci where it then recruits and assists in ATM phosphorylation and similarly activated downstream DNA damage-induced proteins. [32,34,35] Our results demonstrate RAGE as a member of these complexes, suggesting the recruitment of RAGE to the DNA-DSBs in the PE and PTL placentas and a novel role for RAGE in maintaining DNA integrity. To further identify potential molecular interplay between RAGE and DNA-DSB related proteins, DNA-DSBs were induced by CSE or BLM treatment of trophoblast cell lines representing the syncytiotrophoblast (Bewo) and the invasive cytotrophoblast (Sw.71). The induction of DNA-DSBs was confirmed by increased nuclear γ-H2AX in the treated cell lines. More interestingly, DNA damage was further augmented when RAGE neutralizing antibody was added in conjunction with the DNA-DSB inducing treatments. Although the exact mechanisms that control nuclear RAGE expression and its nuclear functions are not known, the RAGE neutralizing antibody studies suggests that perhaps membrane bound activation of RAGE is necessary for subsequent nuclear RAGE-mediated protection from DNA-DSBs. These results portend a possible role for RAGE in genomic protection when DNA damaging stimuli encounter trophoblast cells. Concomitantly, nuclear RAGE protein was also increased in the treated cells as compared to controls. This suggests a possible role for this receptor in maintaining genomic integrity in cell lines in a fashion similar to what was observed in the diseased placentas. Support for a possible role of RAGE in pathways downstream of damage recognition, and at stages when repair is initiated, was further verified in trophoblast cells where RAGE-associated with pATM and MRE11 during CSE or BLM, induced DNA-DSBs.

Defective DNA accumulation at the organ level is known to elicit negative effects on otherwise normal physiological functions. For example, shallow invasion of trophoblast cells is a hallmark of several complicated pregnancies, such as those affected by PE [7]. To examine the correlation between DNA-DSBs and trophoblast invasion, we measured the invasiveness of Sw.71 cytotrophoblasts during CSE or BLM induced DNA-DSBs. We demonstrated that the induction of DNA-DSBs by both CSE and BLM resulted in decreased trophoblast cell invasion, demonstrating the importance of DNA stability in the regulation of trophoblast invasion. Importantly, we showed that neutralization of RAGE significantly hindered trophoblast invasion, a discovery that revealed in vitro support for expanding the role for RAGE to include genomic stabilization during exposure to DNA-DSBs.

Collectively, our results demonstrated the importance of DNA stability in the regulation of placental/trophoblast behavior and invasion. More importantly, we have initiated a line of research that expands the scope of RAGE biology to include nuclear effects that are necessary for identifying DNA-DSBs and preventing notable loss of DNA integrity in the placenta. Further studies are needed to demonstrate specific roles for RAGE when placental cells experience DNA-DSBs. For instance, a natural extension of the current work is to perform a series of analyses that aim to characterize DNA-DSB incidence, recognition, and repair when RAGE expression is targeted. Such in vitro and in vivo research should include the knocking out of the RAGE gene in order to confirm a potential vital role in protecting genomic integrity in the event damaging agents are encountered. This critical research would also confirm to what extent RAGE utilization enhances DNA-DSB repair, in order to slow disease progression.

## 5. Conclusions

The results summarized in the present investigation provide an important initial step in understanding DNA-DSBs and roles for RAGE that could foreshadow new avenues of study with possible therapeutic utility.

## Figures and Tables

**Table 1:** Demographical Data from collected placental samples. Parameters between control and disease placental groups (*n* = 6) were analyzed for statistical significance (*p* < 0.05) using the Kruskal-Wallis test.

	Control	GDM	PTL	PE	P Value
Maternal Age	34 ± 2.96	35 ± 2.8	29 ± 1.6	36 ± 2.2	0.494
Gestational Age (wks)	38 ± 0.02	39 ± 1.6	32 ± 0.5	32 ± 2.3	0.002
Fetal Weight (g)	3498 ± 59	3247 ± 172	2025 ± 139	2025 ± 139	0.002

% C-section/Vaginal 90%/10%.

**Table 2:** List of antibodies used per application.

Antibody	Species	Supplier	Application
RAGE	Goat	R&D (AF1145)	WB, IF
RAGE	Mouse	Abcam (ab89911)	Neutralizing
Phospho-γ-H2AX	Rabbit	Cell Signaling (9718)	WB, IF
Phospho-pATM	Rabbit	Abcam (ab81292)	IP
MRE11	Rabbit	Cell Signaling (4895)	IP

## Data Availability

All data discussed are presented within the article.

## Abbreviations

ATM	activate ataxia-telangiectasia-mutated
CSE	cigarette smoke extract
DDR	DNA damage response
DSBs	double-strand breaks
γ-H2AX	phosphor H2A histone family member X
GDM	gestational diabetes mellitus
HMGB1	high mobility group box 1
HRR	homologous recombination repair
MRE11	double-strand break repair protein MRE11
MRN	MRE11-Rad50-Nbs complex
NHEIJ	non-homologous end joining
PE	preeclampsia
PTL	preterm labor
RAGE	receptors for advanced glycation end-products
Sw.71	Swan 71 first trimester trophoblast cells
IUGR	intrauterine growth restriction
